# Supplementary figures and images for: Estimating internal dissolved methane loading in rivers using a mass balance approach
Source: PeerJ. 2025 Oct 15;13:e20238. doi: 10.7717/peerj.20238 (PMC12535232; doi:10.7717/peerj.20238)

## Width (m)

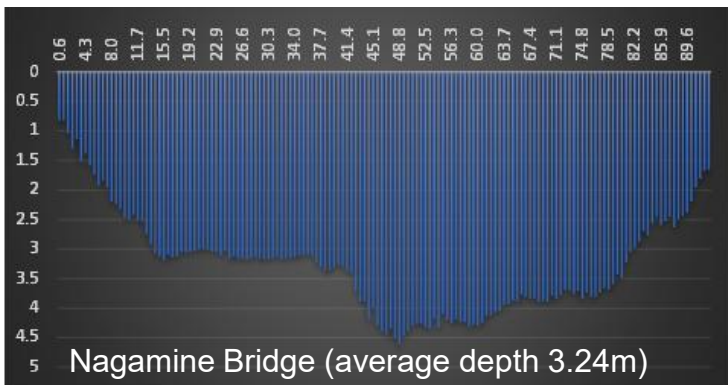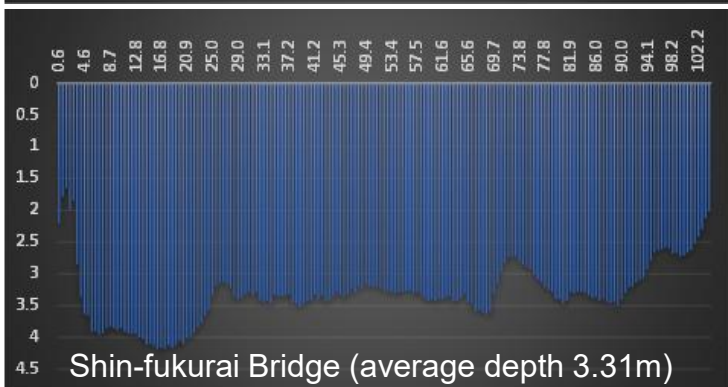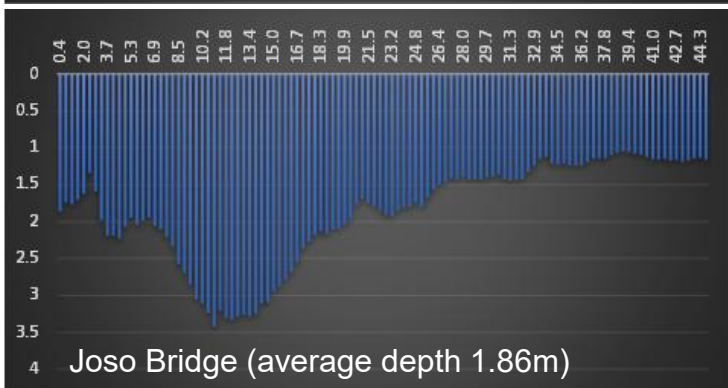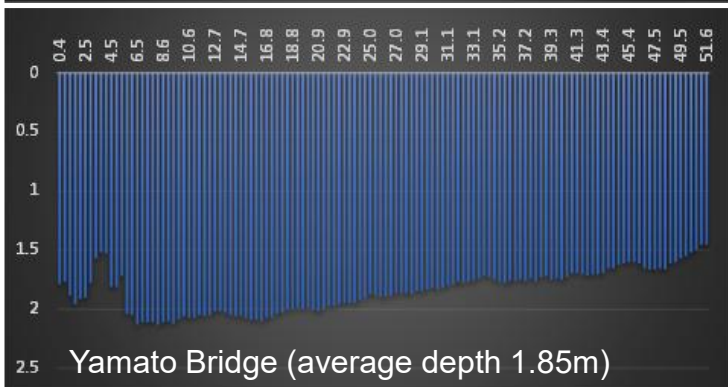

Depth (m)

Supplement: Supplemental Information 2 [file peerj-13-20238-s002.pdf]

● Nagamine ● Shinfukurai ● Joso ● Yamato

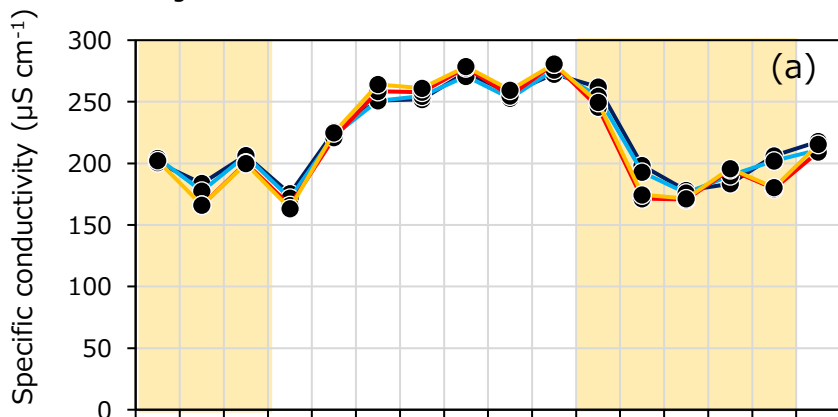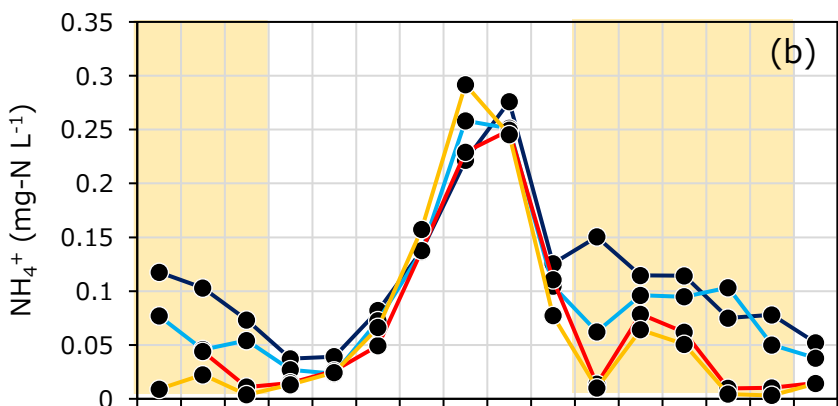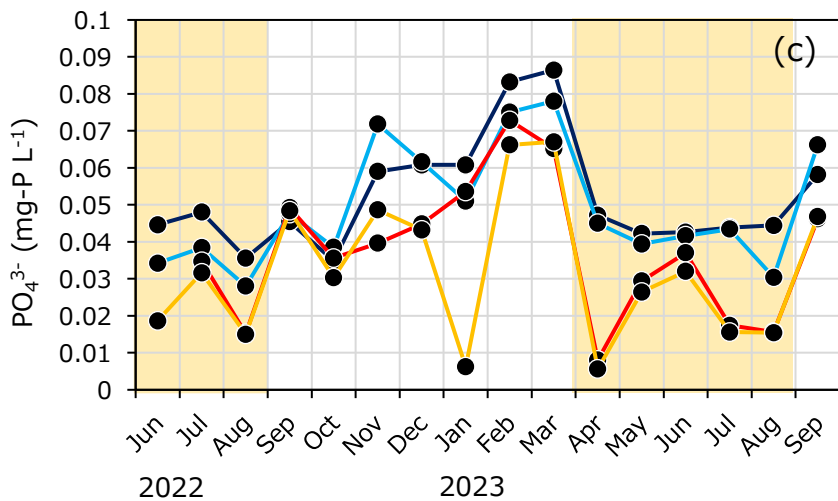

Supplement: Supplemental Information 3 [file peerj-13-20238-s003.pdf]

—●— Nagamine    —●— Shinfukurai    —●— Joso    —●— Yamato

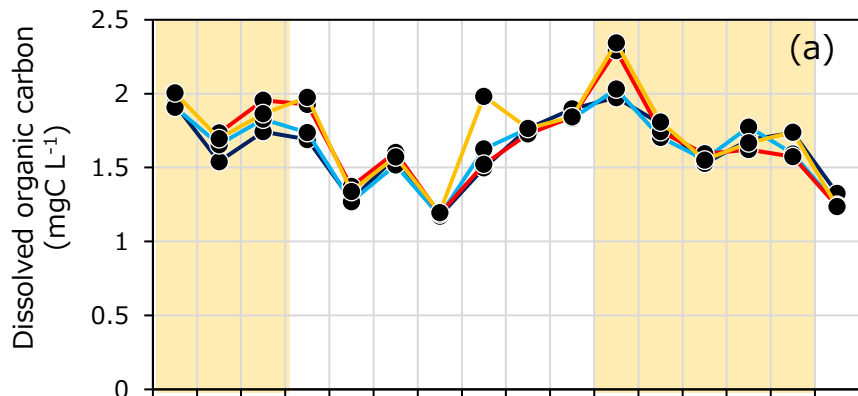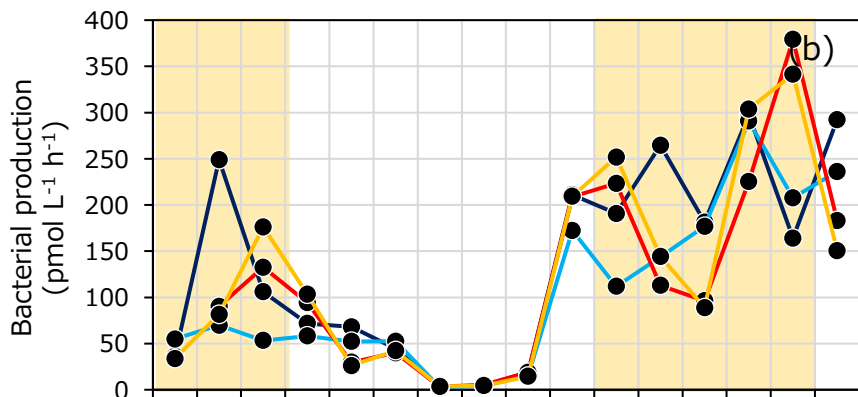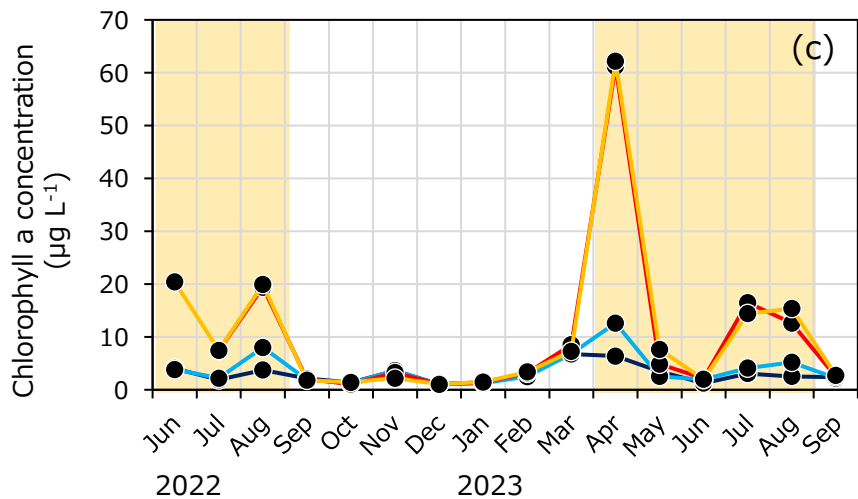

Supplement: Supplemental Information 4 [file peerj-13-20238-s004.pdf]
